# Highly stable peptide adducts from hard keratins as biomarkers to verify local sulfur mustard exposure of hair by high-resolution mass spectrometry

**DOI:** 10.1007/s00204-022-03307-0

**Published:** 2022-05-16

**Authors:** Wolfgang Schmeißer, Markus Siegert, Horst Thiermann, Theo Rein, Harald John

**Affiliations:** 1 Bundeswehr Institute of Pharmacology and Toxicology, Neuherbergstrasse 11, 80937 Munich, Germany; 2 Department of Chemistry, Humboldt-Universität Zu Berlin, Brook-Taylor-Strasse 2, 12489 Berlin, Germany; 3 Max Planck Institute of Psychiatry, Kraepelinstrasse 2-10, 80804 Munich, Germany; 4 Proteros Biostructures GmbH, Bunsenstrasse 7a, 82152 Planegg, Germany

**Keywords:** Hydroxyethylthioethyl-moiety, Organisation for the Prohibition of Chemical Weapons, Peptide biomarker, Protein adducts, Vesicant

## Abstract

In the recent past, the blister agent sulfur mustard (SM) deployed by the terroristic group Islamic State has caused a huge number of civilian and military casualties in armed conflicts in the Middle East. The vaporized or aerolized agent might be inhaled and have direct contact to skin and hair. Reaction products of SM with plasma proteins (adducts) represent well-established systemic targets for the bioanalytical verification of exposure. The SM-derived hydroxyethylthioethyl (HETE)-moiety is attached to nucleophilic amino acid side chains and allows unambiguous adduct detection. For shipping of common blood and plasma samples, extensive packaging rules are to be followed as these matrices are considered as potentially infectious material. In contrast, hair is considered as non-infectious thus making its handling and transportation much less complicated. Therefore, we addressed this matrix to develop a procedure for bioanalytical verification. Following optimized lysis of SM-treated human scalp hair and pepsin-catalyzed proteolysis of adducts of keratin type I and II, microbore liquid chromatography–electrospray ionization high-resolution tandem-mass spectrometry (µLC–ESI MS/HR MS) was used to detect three alkylated keratin-derived biomarker peptides: AE(-HETE)IRSDL, FKTIE(-HETE)EL, and LE(-HETE)TKLQF simultaneously. All bear the HETE-moiety bound to a glutamic acid residue. Protein adducts were stable for at least 14 weeks at ambient temperature and contact to air, and were not affected by washing the hair with shampoo. The biomarker peptides were also obtained from beard, armpit, abdominal, and pubic hair. This is the first report introducing stable local peptide adduct biomarkers from hair, that is easily accessible by a non-invasive sampling process.

## Introduction

Vesicants—a subgroup of chemical warfare agents (CWA)—cause a variety of local and systemic clinical symptoms depending on the dose and time of exposure including blisters and erythema (Ghabili et al. 2010). Sulfur mustard (SM, bis(2-chloroethyl)sulfide, CAS No. 505-60-2) is the most prominent representative of these blister agents. It has been used repeatedly in armed conflicts in the recent past, e.g., in the Syrian Arab Republic and Northern Iraq deployed by the terroristic group Islamic State as well as in the Iran–Iraq war in large scale (John et al. 2019; Sezigen and Kenar 2020). SM is banned by the Chemical Weapons Convention (CWC) which adherence is supervised by the Organisation for the Prohibition of Chemical Weapons (OPCW) (https://www.opcw.org/sites/default/files/documents/CWC/ CWC_en.pdf). To investigate the alleged use of CWA, laboratories designated by the OPCW for the analysis of biomedical samples target specific systemic biomarkers and biotransformation products (Blum et al. 2014; John et al. 2020).

Especially, reaction products (adducts) of SM formed with human proteins such as serum albumin after incorporation are highly useful for biomedical verification due to their long-term traceability for up to several weeks post-exposure (John et al. 2019, 2020; Steinritz et al. 2016; Xu et al. 2014). Such adducts possess the SM-characteristic hydroxyethylthioethyl (HETE)-moiety that is attached to nucleophilic side chains of certain amino acids including, e.g., cysteine, methionine, and glutamic acid (John et al. 2016, 2019; Siegert et al. 2019; Steinritz et al. 2016, 2021). Proteolysis of these protein adducts generates biomarker peptides that can be detected by liquid chromatography–mass spectrometry.

In real case exposure scenarios with vaporized or aerosolized SM, the agent will have direct contact to skin and hair (John et al. 2019; Sezigen and Kenar 2020; Steinritz et al. 2016). Accordingly, the formation of adducts with local proteins is expected (Steinritz et al. 2021). Typical forensic methods make use of hair to detect, e.g., drugs, that either stick to the surface of the hair or have been incorporated into the hair during its growth (Ferreira et al. 2019). However, these methods do not target agent-induced covalent protein modifications and do not detect adducted peptide biomarkers.

Hair predominantly consists of the structural proteins hard keratins (65–95% w/w) (Robbins 2012), that are subdivided into two types. Type I keratins possess a molecular weight (MW) of 40–50 kDa and an isoelectric point (pI) at acidic pH values. Eleven type I keratins are known, which are numbered as K 31–K 40 including K 33A and K 33B (Langbein et al. 2001; Schweizer et al. 2006). Type II keratins possess an MW of 55–65 kDa, a pI at neutral to slightly basic pH values and comprise 6 proteins (K 81–K 86) (Langbein et al. 2001; Schweizer et al. 2006). Keratins are particularly rich in cysteines that form a large number of intra- and intermolecular disulfide bridges (Lubec et al. 1987; Robbins 2012; Robbins and Kelly 1970). This high level of crosslinking is responsible for the structural rigidity and insolubility of hair (Lubec et al. 1987). Accordingly, analysis of human hair proteins requires their initial lysis to reduce and thus cleave disulfide bridges and make the proteins soluble (Adav et al. 2018).

The analysis of covalent hair protein modifications is addressed by only a very limited number of reports so far (Adav et al. 2018; Barthélemy et al. 2012; Grosvenor et al. 2016; Lee et al. 2006; Watson et al. 1998). Desamination (Adav et al. 2018), oxidation (Barthélemy et al. 2012; Grosvenor et al. 2016), mono-, di-, and trimethylation (Lee et al. 2006; Watson et al. 1998), and phosphorylation and ubiquitination (Lee et al. 2006) were reported as posttranslational modifications. In addition, keratin-acetaldehyde adducts were found in murine hair after feeding ethanol (Watson et al. 1998). The formation of SM adducts with soft keratins from skin (van der Schans et al. 2002) and from cultured human epidermal keratinocytes (HEK) (Dillman et al. 2003; Mol et al. 2008) has already been shown but adducts of hard hair keratins with SM or any other CWA have never been described before.

In principle, hair specimens are beneficial for forensic analysis as it is easily accessible in a non-invasive process and not subject to the regulations of the International Air Transport Association (IATA) for potentially infectious material thus not requiring the adherence to complex rules for packaging and shipping (International Air Transport Association 2021; U.S. Department of Labor 2021). Therefore, the aim of this study was to investigate potential keratin adducts in human hair and develop the first micro-liquid chromatography–electrospray ionization high-resolution tandem-mass spectrometry (µLC–ESI MS/HR MS) method for the detection of related local peptide biomarkers for the verification of SM exposure.

## Materials and methods

### Chemicals

Chemicals were purchased from common commercial sources. Pepsin from porcine gastric mucosa was from Sigma-Aldrich (Steinheim, Germany, lot no. SLBQ1670V), 2D-Quant Kit from GE Healthcare (Freiburg, Germany), NaOCl solution for decontamination from Carl Roth (Karlsruhe, Germany), deuterated atropine (d_3_-Atr) from CDN Isotopes (Pointe-Claire, Quebec, Canada), and hair shampoo from a common commercial provider. SM was provided by the German Ministry of Defense and checked in-house for integrity and purity by NMR. Working solutions of SM were prepared in acetonitrile yielding concentrations ranging from 15 µM to 50 mM. Scalp hair was a donation from 7 human non-exposed male and female individuals. Additional beard, abdominal, armpit, and pubic hair was obtained from one male individual. Pooled scalp hair was produced by mixing equal amounts of minced hair from all donors. Sweat was collected from the skin of several volunteers after intense physical activity and purified from particles by ultrafiltration (UF) prior to use (Amicon Ultra-0.5 centrifugal filter unit, 0.5 mL, molecular weight cut-off, MWCO, 10 kDa, Merck Millipore, Billerica, Massachusetts, USA).

### Initial rinsing of hair

Hair was rinsed three times with methanol (70% v/v) and three times with H_2_O prior to air drying (16 h, room temperature, RT). Afterwards, the hair was cut into pieces (2–3 mm length) and stored at RT until use.

### Incubation of hair with SM

Pieces of hair (10 mg in total) were covered with SM working solutions (150 µL) for references (25 mM SM) and standards (diverse SM concentrations) and with neat acetonitrile (150 µL) for blanks and mixed with H_2_O (450 µL, each). After 30 min, the mixture was centrifuged (15,000 RCF, 10 min, 25 °C), the liquid phase was discarded, and hair was washed three times with acetonitrile (200 µL). Subsequently, the hair was dried at RT for 1 h and stored at  – 20 °C prior to use. Scalp hair from seven individuals and pooled hair as well as scalp, beard, abdominal, armpit, and pubic hair from one individual was incubated (*n* = 3, each).

### Lysis of hair

Lysis of hair was performed based on the Shindai method (Fujii et al. 2013). In brief, following the standard protocol, lysis buffer (700 µL, 20 mM Tris–HCL, 2.6 M thiourea, 5 M urea, 20 mM dithiothreitol, pH 9.0) was added to hair (10 mg) and shaken constantly for 4 h (50 °C). After centrifugation (15,000 RCF, 10 min, 25 °C), 500 µL was transferred into a reaction vial followed by carbamidomethylation with iodoacetamide (26 µL, 1 mM) under gentle shaking in the dark (1 h, 50 °C). During method optimization, different periods of lysis were tested (0.25 h, 0.5 h, 1 h, 2 h, 4 h, 8 h, 12 h, 16 h, 20 h, and 24 h).

### Proteolysis of keratins

Following the standard protocol, the reaction mixture obtained after lysis and carbamidomethylation (500 µL) was subjected to UF (15,000 RCF, 10 min, 35 °C). The retentate was washed 2-times by UF after the addition of H_2_O (300 µL) and subsequently two times after the addition of formic acid (FA, 300 µL, 10% v/v). Proteolysis of proteins in the retentate was performed by adding pepsin solution (100 µL, 2.5 mg/mL in FA 10% v/v) and FA (300 µL, 10% v/v) under constant shaking (2 h, 42 °C). During method optimization, diverse periods for proteolysis were tested (1.5 min, 3 min, 5 min, 10 min, 15 min, 30 min, 45 min, 60 min, 90 min, 120 min, 150 min, 180 min, 210 min, 240 min, 270 min, and 300 min). After UF (15,000 RCF, 15 min, 20 °C), 30 µL of the filtrate was mixed with 60 µL d_3_-Atr solution (3 ng/mL in FA 0.5% v/v) prior to µLC–ESI MS/HR MS analysis. Measurements were performed using either an Orbitrap system working in data-dependent tandem-MS mode (ddMS2) or a quadrupole time-of-flight (TOF) system operating in product ion scan (PIS) mode.

### μLC-ESI MS/HR MS (ddMS2) analysis

For identification of alkylated peptides by µLC–ESI MS/HR MS, the ddMS2 mode was carried out using a QExactive plus Orbitrap mass spectrometer (Thermo Scientific, Bremen, Germany) working with positive ESI. The common instrumental equipment and software used were the same as described recently (Blum et al. 2020; John et al. 2021). An Acquity HSS T3 column (50 × 1.0 mm I.D., 1.8 µm, 100 Å, Waters, Eschborn, Germany) protected by a precolumn (Security Guard™ Ultra Cartiges UHPLC C18 peptide 2.1 mm I.D., Phenomenex, Aschaffenburg, Germany) was used for chromatographic separation of 20 µL sample volume at 30 °C with a linear gradient of solvent A (0.05% v/v FA) and solvent B (acetonitrile/H_2_O 80:20 v/v, 0.05% v/v FA) at a flow of 30 µL/min: t [min]/B [%]: 0/0; 3/0; 35/40; 35.5/95; 39.5/95; 40/0.

The ddMS2 approach was comprised of an initial full MS scan followed by MS/MS of the ten most intense ions (top 10) (John et al. 2021). The following parameters were applied for full MS: spray voltage 3.5 kV, capillary temperature 250 °C, sheath gas flow rate 23 arbitrary units (a.u.), mass spectrometric resolution at full width at half maximum (fwhm) 70,000; automatic gain control (AGC) target 3.0 × 10^6^, maximum injection time (MIT) 100 ms, and scan range *m/z* 200—*m/z* 1500. For ddMS2, the following settings were used: fwhm 17,500, AGC target 1.0 × 10^5^, MIT 150 ms, isolation window 1.6 m*/z*, normalized collision energy (NCE) 30; minimum AGC target 3.0 × 10^3^, intensity threshold 2.0 × 10^4^, charge inclusion 2–7, and dynamic exclusion 30 s.

### Data interpretation

MS/MS spectra of peptides fragmented by ddMS2 were analyzed by the Proteome Discoverer 2.1 software (Thermo Scientific) and measurements were matched to a database containing amino acid sequences of all hair keratins (K 31–K 40, K 81–K 86) taken from the UniProt database considering the SM-derived HETE-attachment (+ 104.030 Da at Cys, Asp, Glu, His, Met) and carbamidomethylation (+ 57.021 Da at Cys) using the Sequest HT algorithm. Peptides proposed to bear SM alkylation were manually checked for the presence of a signal at *m/z* 105.037 representing the cleaved [HETE]^+^-moiety. The identity of proposed alkylated peptides was subsequently confirmed by µLC–ESI MS/HR MS analysis in PIS mode.

### μLC–ESI MS/HR MS (PIS) analysis

For µLC–ESI MS/HR MS (PIS) analysis, a microLC 200 pump (Eksigent Technologies LLC, Dublin, CA, USA) on-line coupled to a TripleTOF 5600^+^ mass spectrometer (TT5600^+^, ABSciex, Darmstadt, Germany) was used as described recently (Schmeißer et al. 2021). Chromatographic separation (30 °C, 30 µL/min) of 20 µL sample was performed on an Acquity HSS T3 (100 × 1.0 mm I.D., 1.8 µm, 100 Å, Waters), protected by a precolumn as described above. After 5 min of equilibration under starting conditions, a gradient of solvent A and solvent B was applied: t [min]/B [%]: 0/5; 4/29; 12.5/32; 16/50; 16.5/95; 18.5/95; 19/5; 20/5.

Product ions of the double-protonated alkylated peptides AE(-HETE)IRSDL, FKTIE(-HETE)EL, and LE(-HETE)TKLQF were obtained after collision-induced dissociation (CID) and monitored in the mass range from *m/z* 50 to *m/z* 950 with an accumulation time of 100 ms, each. Individual masses of precursor ions and of the two most intense product ions (qualifier I and qualifier II) are listed in Table 1. The following MS parameters were the same for the three alkylated peptides: collision energy spread (CES) 3 V, curtain gas (CUR) 2.07 × 10^5^ Pa (30 psi), heater gas (GS1) 2.76 × 10^5^ Pa (40 psi), turbo ion spray gas (GS2) 3.45 × 10^5^ Pa (50 psi), ion release delay (IRD) 67 ms, ion release width (IRW) 25 ms, ion spray voltage floating (ISVF) 5000 V, and temperature (TEM) 200 °C.

**Table 1:** MS parameters for the detection of hair keratin-derived biomarkers and internal standard

Compound	Precursor ion	*m/z*	DP [V]	CE [V]	Qual I *m/z*	Qual II *m/z*	Peak area ratio Qual II/Qual I [%]
AE(-HETE)IRSDL	[M + 2H]^2+^	454.2	50	24	803.4258	105.0369	60.2
FKTIE(-HETE)EL	[M + 2H]^2+^	492.3	40	22	879.4822	105.0369	68.7
LE(-HETE)TKLQF	[M + 2H]^2+^	491.8	50	22	878.4982	105.0369	86.7
d_3_ -Atr	[M + H]^+^	293.2	100	42	127.1309	93.0699	51.0

Settings were used for µLC–ESI MS/HR MS (PIS) analysis on a TT5600^+^ system. d3-Atr (triple deuterated atropine) was used as internal standard. For each peptide, the qualifier I (Qual I) represents the [M + H] lost HETE^+^ ion (single protonated peptide backbone after the loss of the [HETE]^+^-moiety), whereas Qual II is the cleaved [HETE]^+^-ion. CE: collision energy, DP: declustering potential, HETE: hydroxyethylthioethyl-moiety

### Selectivity of the µLC–ESI MS/HR MS (PIS) method and interindividual differences in biomarker yield

Blank and reference hair from 7 donors (scalp, 10 mg, *n* = 3, each) as well as blank and reference scalp, beard, abdominal, armpit, and pubic hair (10 mg, *n* = 3, each) from one individual were prepared according to the standard protocol and analyzed by µLC–ESI MS/HR MS (PIS) to monitor any interference in blanks and to compare individual biomarker peak areas in references.

### Limit of identification of biomarker peptides

Pooled scalp hair (10 mg) was incubated with SM working solutions (*n* = 3) to generate standards corresponding to SM concentrations of 12 µM, 24 µM, 48 µM, 96 µM, 190 µM, 390 µM, 780 µM, 1.56 mM, 3.125 mM, 6.25 mM, 12.5 mM, 25 mM, and 50 mM. Following the standard protocol, relative peak area ratios of the respective qualifier II to qualifier I of the three biomarker peptides (Table 1, given in percent) were calculated. The limit of identification (LOI) was defined as the lowest concentration of SM at which all measurements of the triplicate met the area ratios obtained from pooled hair reference.

### Stability of biomarker peptides in the autosampler

To evaluate the stability of the three biomarker peptides stored in the autosampler at 15 °C, pooled hair reference (scalp, 10 mg) was prepared according to the standard protocol and analyzed by µLC–ESI MS/HR MS (PIS) hourly within a period of 24 h. Biomarker peak areas were determined to monitor relative concentration–time profiles.

### Time-dependent adduct formation in the presence of water

Pooled scalp hair (10 mg) was mixed with SM working solution (150 µL, 25 mM) and additionally with either H_2_O or acetonitrile (450 µL, each). The liquid phase was discarded after 1 min, 2 min, 5 min, 10 min, 15 min, 20 min, 25 min, 30 min, 60 min, 90 min, and 120 min (tested as separate incubation mixtures, n = 3, each). Immediately afterwards, acetonitrile (200 µL) was added to the hair for vortex-mixing. Removal of the liquid phase and addition of another acetonitrile portion (200 µL) was repeated five times. Following standard lysis and proteolysis, the samples were analyzed by µLC–ESI MS/HR MS (PIS) to monitor the relative time-dependent concentrations of all three biomarker peptides.

### Influence of sweat on adduct formation

Pooled hair (scalp, 10 mg) was incubated with SM either in the presence of H_2_O (450 µL, *n* = 3) or in the presence of sweat (450 µL, *n* = 3). Subsequent sample preparation and analysis were done according to the standard protocol to determine the peak areas of the three biomarker peptides.

### SDS-PAGE of lysed hair and in-gel proteolysis with pepsin

After lysis of blank and pooled hair reference (scalp, 10 mg, *n* = 3, each), the supernatant containing 30 µg protein (quantified by 2D-Quant Kit) was separated by SDS-PAGE (NuPAGE™ 4–12% bis Tris Gel, Thermo Fisher Scientific, Waltham, MA, USA). Subsequent staining with Coomassie Brilliant Blue (CBB) and in-gel proteolysis (2 h) were carried out according to common protocols (Thermo Fisher Scientific 2021) but using pepsin (2.5 mg/mL FA 10% v/v) instead of trypsin. The three biomarker peptides were detected by µLC–ESI MS/HR MS (PIS).

### Stability of alkylated keratins in hair during storage

To characterize the stability of alkylated keratins in hair, pooled hair reference (scalp, 10 mg, *n* = 63) was stored under the following conditions:  – 20 °C in the dark and exclusion of air (condition I, *n* = 21); 25 °C in the dark and exclusion of air (condition II, *n* = 21) and 25 °C with daylight and contact to air (condition III, *n* = 21). Hair was analyzed weekly (*n* = 3 per condition) according to the standard protocol over a period of 7 weeks monitoring the relative concentration–time profiles of all three biomarker peptides. In addition, the period of analysis for condition III was prolonged for another 7 week period (14 weeks in total).

### Stability of alkylated keratins in hair during wash cycles with shampoo

Pooled hair reference (scalp, 10 mg) was mixed with 500 µL of a water–shampoo mixture (H_2_O/shampoo 4:1 v/v) and shaken for 5 min (40 °C). After centrifugation (15,000 RCF, 10 min, 25 °C), the supernatant was removed and the procedure was repeated five times with 500 µL H_2_O instead of the water–shampoo mixture. Finally, the hair was dried at RT for 24 h. This entire wash cycle with shampoo and water was repeated on 4 consecutive days. After each cycle, samples (*n* = 3) were processed according to the standard protocol and analyzed by µLC–ESI MS/HR MS (PIS). Peak areas of all biomarker peptides were compared to those of references not subjected to any wash cycle.

### Safety considerations

SM is a highly toxic blister agent. Only trained personnel wearing laboratory protective clothes should handle it under the fume hood. Decontamination of all materials that have come into contact with SM is mandatory. Decontamination should be done by submerging the material into alkaline NaOCl solution and leaving it in the solution for several hours.

## Results and discussion

Blood and plasma, that are the most common specimens used for verification analysis, are often considered as potentially infectious material if no further information about the health of the donor or the status of the sample itself is available (International Air Transport Association 2021). Accordingly, strict rules for shipment have to be followed with respect to triple packaging. The use of a leak-proof primary receptacle, a leak-proof secondary receptacle containing absorbent material and a clearly labelled rigid outer package shall prevent from the discharge of the sample and allow immediate identification by its UN specification number e.g., UN 3373 (biological substance, category B). If there is a minimal likelihood that pathogens are present, such blood and plasma samples might be categorized and labelled as “exempt human specimens”, but they still require triple packaging. In contrast, hair is in principle considered as non-infectious material (U.S. Department of Labor 2021) and can easily be transported even in cabin luggage and presented at security checkpoints in clear containers (such as zip-lock bags) making passage of samples much easier. In addition, it is easily accessible by a non-invasive process. Therefore, hair represents a highly valuable alternative specimen for the verification of SM exposure and was thus addressed in the present study. Initially, the formation of SM adducts had to be proven after lysis and proteolysis.

### Identification of alkylated peptides by µLC–ESI MS/HR MS

Alkylation of proteins by SM in aqueous media typically yields an attached HETE-moiety still present in biomarker peptides after proteolysis (John et al. 2016, 2019, 2020; Siegert et al. 2019; Steinritz et al. 2021). MS fragmentation of these adducted peptides typically produces the [HETE]^+^-product ion at *m/z* 105.037 (John et al. 2016, 2019; Siegert et al. 2019; Steinritz et al. 2016, 2021) that thus represents a diagnostic ion indicating alkylation. To identify so far unknown alkylated peptides, hair incubated with SM was lysed, proteolyzed with pepsin, and analyzed by µLC–ESI MS/HR MS (ddMS2) using an Orbitrap mass spectrometer for optimum mass resolution (Blum et al. 2020; John et al. 2021). Even though the use of trypsin would have simplified the prediction of cleaved peptides, pepsin was used, because the acidic proteolysis conditions prevent the hydrolysis of alkylated amino acids, therefore optimizing adduct yield.

Precursor ions, that yielded the [HETE]^+^-ion, were correlated to keratin amino acid sequences known from UniProt database entries. For mass spectrometric structural confirmation of proposed peptide adducts selective µLC–ESI MS/HR MS (PIS) analyses were performed considering different charge states of the proposed precursor ions. Optimum properties in terms of signal intensity and chromatographic behavior were found for the three double-protonated alkylated heptapeptides AE(-HETE)IRSDL ([M + 2H]^2+^, *m/z* 454.2), FKTIE(-HETE)EL ([M + 2H]^2+^, *m/z* 492.3) and LE(-HETE)TKLQF ([M + 2H]^2+^, *m/z* 491.8) all bearing an alkylated glutamic acid residue (Table 1). The peptide AEIRSDL derived either from K 31 (UniProtKB Q15323) or K 33A/B (UniProtKB O76009/Q14525). FKTIEEL was produced from either K 31, K 33A/B or K 34 (UniProtKB O76011) and LETKLQF originated either from K 81 (UniProtKB Q14533) or K 83 (UniProtKB P78385) or K 86 (UniProtKB O43790). These assignments were also supported by SDS-PAGE analysis as described below (section: SDS-PAGE of lysed hair and in-gel proteolysis with pepsin).

### Monitoring of biomarker peptides by µLC–ESI MS/HR MS (PIS)

**Fig. 1:**
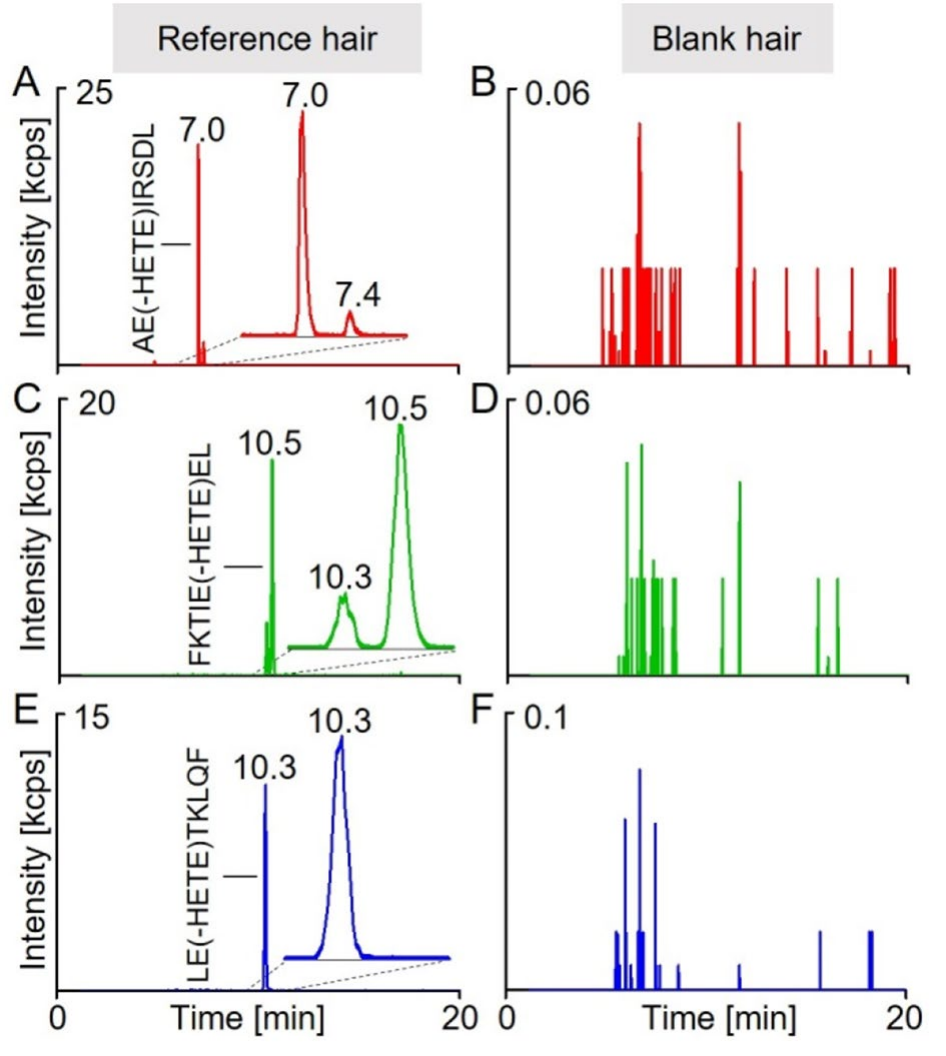
µLC–ESI MS/HR MS (PIS) detection of biomarker peptides of sulfur mustard exposure of hair. μLC–ESI MS/HR MS (PIS) analysis of pooled hair reference exposed to sulfur mustard (25 mM, left column) and blank hair (right column) after lysis and proteolysis with pepsin. For reasons of clarity, only the individual mass traces of the most intense product ions (qualifier I) are depicted. **A**, **B** AE(-HETE)IRSDL at *t*_R_ 7.0 min, *m/z* 454.2 > *m/z* 803.426. **C**, **D** FKTIE(-HETE)EL at *t*_R_ 10.5 min, *m/z* 492.3 > *m/z* 879.482, and **E**, **F** LE(-HETE)TKLQF at *t*_R_ 10.3 min, *m/z* 491.8 > *m/z* 878.498. Product ion traces were extracted with ± 0.005 Da. **A** The additional peak at *t*_R_ 7.4 min corresponded to a positional isomer of AE(-HETE)IRSDL bearing the HETE-moiety most presumably attached to the aspartic acid residue. **C** The peak at *t*_R_ 10.3 min corresponded to a ^13^C_1_-variant of LE(-HETE)TKLQF exhibiting nearly identical precursor and product ion masses as FKTIE(-HETE)EL. HETE: hydroxyethylthioethyl-moiety

**Fig. 2:**
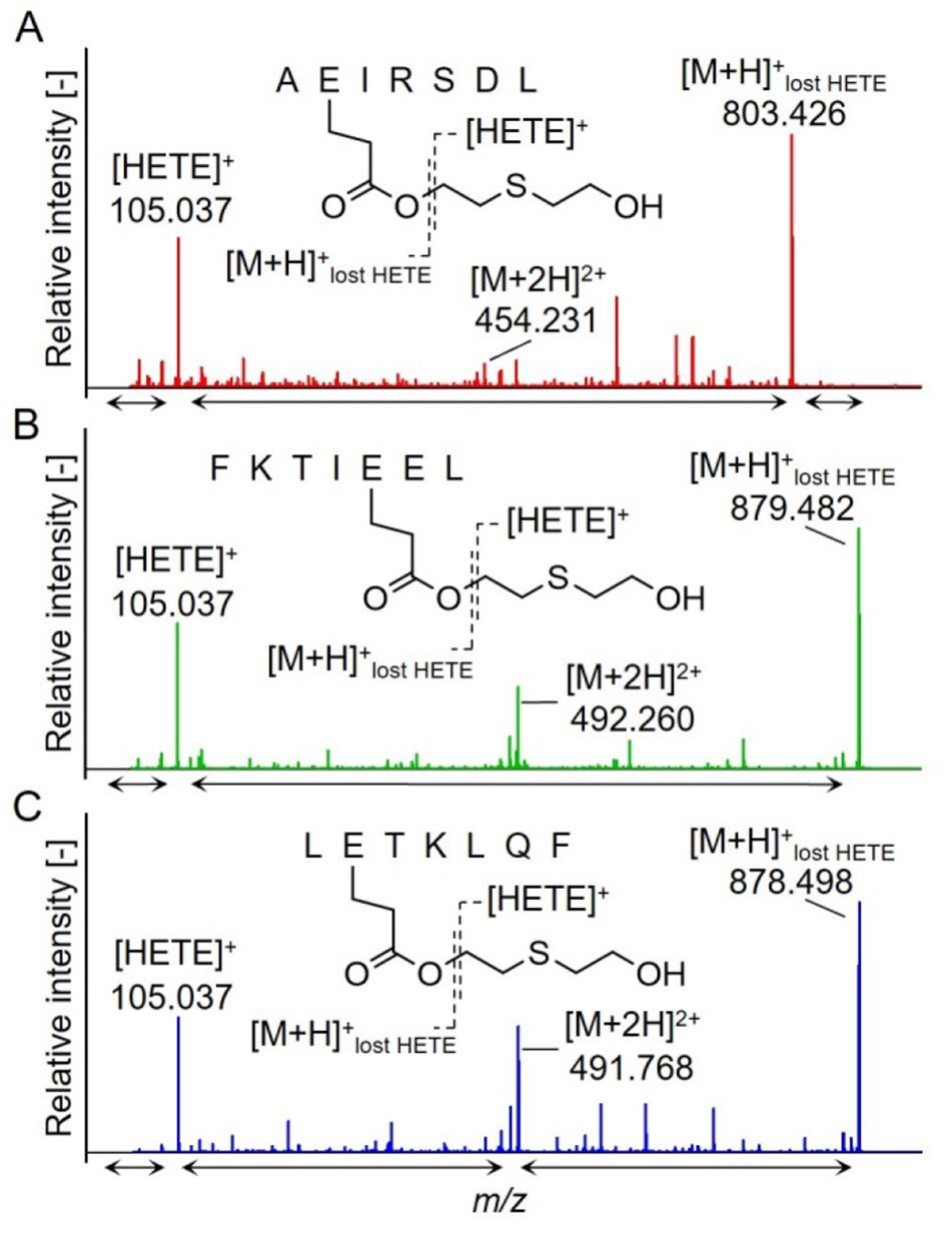
Product ion spectra of biomarker peptides. Pooled hair reference exposed to sulfur mustard (25 mM) was lysed, proteolyzed with pepsin, and analyzed by µLC–ESI MS/HR MS (PIS). Spectra were extracted from chromatographic peaks (Fig. 1) and the most prominent product ions obtained from the double-protonated precursor ions [M + 2H]^2+^ are labelled. **A** AE(-HETE)IRSDL. **B** FKTIE(-HETE)EL. **C** LE(-HETE)TKLQF. [M + H]^+^_lost HETE_ represents the single protonated peptide backbone after the loss of the hydroxyethylthioethyl [HETE]^+^-moiety. All product ions showed a mass deviation ≤ 10 ppm, when compared to their theoretical mass (Table SI 1-SI 3). For better visualization of less abundant product ions not labelled in this figure, their signal intensities were multiplied by a factor of 3 in the marked ranges ( ↔)

A triple TOF mass spectrometer was used to establish a µLC–ESI MS/HR MS (PIS) method for biomarker analysis (Fig. 1). Mass spectrometric parameters of the method (collision energy, declustering potential, ISVF) were optimized for the detection of the individual product ions (qualifier I and qualifier II, Fig. 1; Table 1) with maximum intensity. For all three peptides, the respective most prominent product ion was the single charged peptide backbone [M + H]^+^ after loss of HETE ([M + H]^+^_lost HETE_, qualifier I) resulting from the loss of the [HETE]^+^-moiety (qualifier II, Table 1) as illustrated in Fig. 2.

AE(-HETE)IRSDL: The extracted ion chromatogram (XIC) of qualifier I (*m/z* 803.426, Fig. 1A) showed a major narrow peak at the retention time (t_R_) of 7.0 min and a much smaller well separated peak at *t*_R_ 7.4 min. In addition to qualifier I and II, the MS/MS spectrum of the first eluting compound showed a large number of product ions with minor intensity representing the b- and y-series with and without the attached HETE-moiety (Fig. 2A, Table SI 1). The presence of the alkylated a_2_- (*m/z* 277.122) and alkylated b_2_-ion (*m/z* 305.117) unambiguously proved the site of alkylation at the glutamic acid residue (Table SI 1). The latter ions were not present in the MS/MS spectrum of the second eluting compound (*t*_R_ 7.4 min, data not shown). Nevertheless, the peptide was also identified as alkylated AEIRSDL. Its fragmentation yielded an alkylated y_5_-ion (*m/z* 707.375) documenting alkylation at one of the five C-terminal amino acid residues. No additional product ions containing the HETE-moiety were found, thus preventing the identification of the exact alkylation site. It appears most likely that the side chain of aspartic acid was alkylated. Accordingly, we herein introduce only the first eluting unambiguously identified adduct AE(-HETE)IRSDL as a biomarker peptide. No interference was detected in blank hair (Fig. 1B), thus documenting highest selectivity of the method.

FKTIE(-HETE)EL: The XIC of qualifier I (*m/z* 879.482) showed one peak at *t*_R_ 10.3 min and a second one at *t*_R_ 10.5 min (Fig. 1C). Based on the respective MS/MS data (Fig. 2B, Table SI 2), the larger second peak (*t*_R_ 10.5 min) correlated to FKTIE(-HETE)EL. The presence of the alkylated y_3_-ion (*m/z* 494.217) indicated the alkylation of the glutamic acid residue at position 5 in the heptapeptide (Table SI 2). This identification was also supported by the absence of an ion at *m/z* 365.174 that might have been indicative for the alkylation at the alternative glutamic acid residue at position 6 (alkylated y_2_-ion).

The peptide of the smaller peak (*t*_R_ 10.3 min) was identified by MS/MS as LE(-HETE)TKLQF. The simultaneous detection of both alkylated peptides was due to the fact that the exact so-called ^12^C-monoisotopic mass of both peptides differs by only 0.984 Da. Therefore, the masses of the double charged precursor (*m/z* 492.259) and single charged qualifier I (*m/z* 879.482) of FKTIE(-HETE)EL were nearly identical to the corresponding masses of the ^13^C_1_-variant of LE(-HETE)TKLQF (m/z 492.269 and m/z 879.501). Accordingly, the latter adduct was also subjected to fragmentation and its product ions were detected. However, the ^13^C_1_-variant did not interfere with FKTIE(-HETE)EL due to robust chromatographic baseline separation of both peaks, the excellent reproducibility of retention times (± 0.03 min), as well as their stable chromatographic resolution (*R* = 1.8, ∆*t*_R_ = 0.2 min) observed during the entire period of method development and validation. Furthermore, no interference was found in blank hair (Fig. 1D) thus making FKTIE(-HETE)EL a useful biomarker.

LE(-HETE)TKLQF: The XIC of qualifier I (*m/z* 878.498) showed a single peak at t_R_ 10.3 min (Fig. 1E). No interference was found in blank hair (Fig. 1F), thus proving excellent selectivity of this biomarker also (Table SI 3).

### SDS-PAGE of lysed hair and in-gel proteolysis with pepsin

**Fig. 3:**
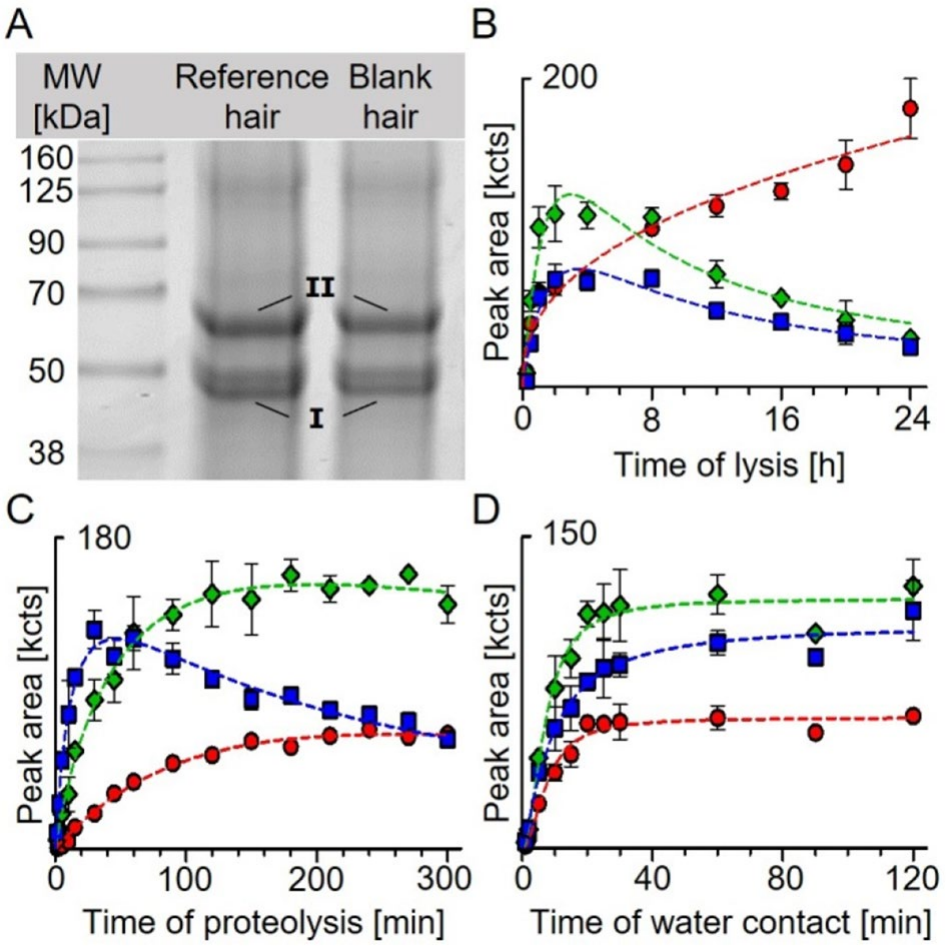
Formation of biomarker peptides while sample preparation. **A** SDS-PAGE of proteins from lysed reference (2nd lane) and blank hair (3rd lane) stained with Comassie Brilliant Blue. I and II mark bands of type I and type II keratins. The peptides AE(-HETE)IRSDL and FKTIE(-HETE)EL derived from the 45 kDa band (I), whereas LE(-HETE)TKLQF was obtained from the 60 kDa band (II). **B** Formation of biomarker peptides during lysis of reference hair exposed to 25 mM sulfur mustard. After indicated periods, samples (*n* = 3) were proteolyzed for 2 h with pepsin and analyzed. **C** Formation of biomarker peptides during proteolysis of reference hair. At indicated time points, aliquots (*n* = 3) were analyzed. **D** Formation of biomarker peptides depending on the contact time of SM-incubated hair with water. At distinct time points (*n* = 3), the supernatant was discarded and hair was washed with acetonitrile. After lysis and proteolysis, analysis was performed. Depicted peak areas of the qualifier I (Table 1) of AE(-HETE)IRSDL (red filled circles), FKTIE(-HETE)EL (green filled diamonds), and LE(-HETE)TKLQF (blue filled squares) were determined by µLC–ESI MS/HR MS (PIS) analysis. Data points represent the mean and SD of triplicate analysis, each

The CBB-stained SDS-PAGE of lysed hair showed one prominent band at 45 kDa (Fig. 3A, I) and another one at 60 kDa (Fig. 3A, II) for both reference (Fig. 3A, 2nd lane) and blank hair (Fig. 3A, 3rd lane). In-gel proteolysis with pepsin revealed the presence of AE(-HETE)IRSDL and FKTIE(-HETE)EL (45 kDa band) and of LE(-HETE)TKLQF (60 kDa band) in the reference documenting their origin from type I (40–50 kDa) and type II (55–65 kDa) hair keratins (Deb-Choudhury et al. 2016). No bands indicating crosslinked keratins were found in contrast to linkage between soft keratin 5 and 14 described earlier in HEK-cells (Dillman et al. 2003). No alkylated peptides were detected in the corresponding bands of blank hair indicating the absence of interferences and documenting the selectivity of biomarker analysis.

### Lysis of hair

During lysis, disulfide bridges were reduced and the complex structure of linked and folded hair proteins destroyed. Therefore, a large amount of hair proteins was dissolved as required for peptide biomarker generation. The time for lysis was varied between 0.25 h and 24 h to define a reasonable period considering both high biomarker yield and best suitability for the laboratory workflow. The peak areas of the three biomarker peptides increased rapidly within the first 2 h (Fig. 3B). Whereas the concentration of AE(-HETE)IRSDL (red filled circles, Fig. 3B) continued to increase afterwards, those of FKTIE(-HETE)EL (green filled diamonds, Fig. 3B) and LE(-HETE)TKLQF (blue filled squares, Fig. 3B) decreased continuously. Accordingly, for the standard protocol, 4 h were selected for lysis providing high concentrations of each biomarker best suited for simultaneous analysis.

### Proteolysis of alkylated keratins

During proteolysis of alkylated keratins derived from reference hair, the concentrations of AE(-HETE)IRSDL (red filled circles, Fig. 3C) and FKTIE(-HETE)EL (green filled diamonds, Fig. 3C) reached a plateau after approx. 130 min. In contrast, LE(-HETE)TKLQF (blue filled squares, Fig. 3C) reached its maximum after about 35 min and continuously decreased afterwards. This was most presumable due to the progressing proteolysis of the peptide rather than to hydrolysis of the HETE-moiety as under acidic conditions applied esters of glutamic acid are known to be stable (John et al. 2019). This assumption was also supported by the stability of AE(-HETE)IRSDL and FKTIE(-HETE)EL (Fig. 3C) which both also contain an alkylated glutamic acid residue. For the standard protocol, a 120 min incubation period was set, providing high concentrations for all three biomarker peptides.

### Selectivity of the µLC–ESI MS/HR MS (PIS) method and interindividual differences in biomarker yield

**Fig. 4:**
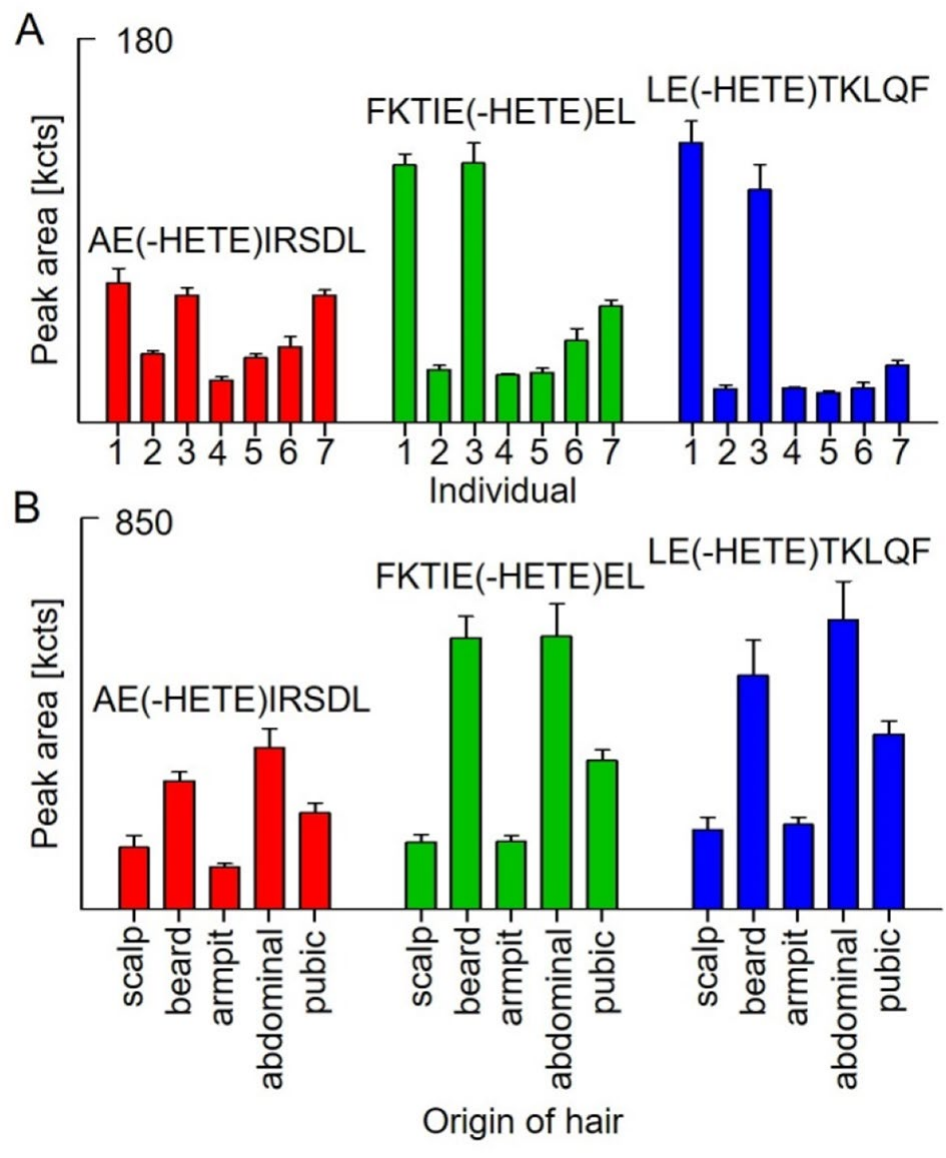
Individual yield of biomarker peptides derived from human hair keratins. Biomarker peptides were analyzed by µLC–ESI MS/HR MS (PIS) after lysis and proteolysis with pepsin of reference hair exposed to 25 mM sulfur mustard. **A** Scalp hair taken from seven human individuals (1–7). **B** Hair from indicated parts of the body of one male individual. Peak areas resulted from extracted ion chromatograms of the qualifier I (± 0.005 Da) of AE(-HETE)IRSDL (red bars), FKTIE(-HETE)EL (green bars) and LE(-HETE)TKLQF (blue bars). Bars represent mean and SD from triplicate analysis, each. HETE: hydroxyethylthioethyl-moiety

No interferences were recorded for qualifier I and qualifier II of all biomarker peptides in blank hair taken from 7 individuals as exemplarily shown in Fig. 1 (right panel), thus documenting highest selectivity of the method. The yield of the three biomarker peptides resulting from SM-incubated hair from 7 individuals (*n* = 3) varied noticeably (Fig. 4A). These differences were most presumable due to the variable composition of human hair consisting of 65–95% w/w keratin (Robbins 2012) as well as to the keratin-dependent extent of intermolecular disulfide crosslinks affecting the efficacy of lysis. However, alkylated peptides were found in high concentrations in the hair of each individual thus documenting the suitability of these biomarkers.

In addition to scalp hair, all three biomarker peptides were also detected in SM-exposed hair taken from other parts of the body (beard, armpit, abdominal and pubic area) (Fig. 4B). The respective yields varied tremendously (Fig. 4B). This phenomenon might have been due to different amounts of keratin or a hair structure-dependent protein yield after lysis. However, the method was shown to be applicable to these hair samples as well and provided reproducible biomarker yields for each origin (RSD ≤ 19%). In principle, this adaptability is highly important when considering that warm and moist areas of the body are typically heavily affected by SM as shown especially for armpit and pubic area (John et al. 2019; Sezigen and Kenar 2020; Steinritz et al. 2016).

### Stability of biomarker peptides in the autosampler

All three biomarker peptides did not show any degradation (RSD ≤ 9.1%) within the 24 h test period, thus documenting excellent stability best suited for analysis of large sample sets (data not shown).

### LOI of biomarker peptides

Hair was incubated with SM in the presence of water yielding concentrations ranging from 3 µM to 12.5 mM. Peak areas of the individual qualifier I showed linear correlations (*R*^2^ ≥ 0.994) between 6 µM and 12.5 mM SM for all biomarkers, thus documenting dose-dependent and reproducible alkylation of hair keratins (data not shown). LOI values obtained from 10 mg hair were as follows: AE(-HETE)IRSDL and FKTIE(-HETE)EL both 190 µM and LE(-HETE)TKLQF 47.5 µM. The low dimension of the LOI documents that only minimum amounts of SM are required for adduct formation and detection. Accordingly, the method is highly suited for real case exposure scenarios where vapor or aerosols of undiluted SM (8 M as liquid) will cover the hair of victims.

### Time-dependent adduct formation in the presence of water

After incubation of hair with SM in the absence of water, no biomarker peptides were detected, independent of the incubation time. In contrast, in the presence of water, the yields of all biomarker peptides increased rapidly within the first 20 min before reaching a plateau, thus indicating an essential role of water for adduct formation (Fig. 3D). The presence of water might be a prerequisite for both the formation of the episulfonium ion representing the highly reactive electrophilic intermediate of SM (John et al. 2020) as well as the transportation of SM into the hair. Hair may absorb up to 32% w/w water depending on the relative humidity of ambient air (Robbins 2012) supporting alkylation. In real cases of SM exposure, water will be present in, e.g., sweaty hair and during decontamination and hair washing, thus inducing adduct formation with SM that still sticks to the hair making our method highly suited for verification.

### Influence of sweat on adduct formation

In the presence of sweat during incubation of hair with SM, resulting biomarker concentrations were reproducible (RSD ≤ 9%, *n* = 3) and about twice as high as obtained with pure water. The higher yield was most presumably due to the salty sweat matrix (1 g minerals/L) (Montain et al. 2007) minimizing the rate of hydrolysis of SM that occurs as a concurrent reaction to alkylation. The period of half-change of SM in H_2_O at 25 °C is about 6 min, whereas the time for hydrolysis in salt water is prolonged by a factor of 3.5 (Bizzigotti et al. 2009). These results support the suitability of our procedure for real cases of sweat-wetted hair exposure.

### Stability of alkylated keratins in hair during storage and wash cycles with shampoo

The concentration of all three biomarker peptides was constant (RSD ≤ 19%) over the entire storage period of hair of 7 weeks under different conditions, thus documenting no degradation of keratin adducts (data not shown). Even a prolonged storage time of another 7 weeks (14 weeks in total) under condition III (25 °C, daylight, contact to air) did not provoke any degradation. Furthermore, 5 wash cycles with the use of shampoo did not affect the biomarker yield (RSD ≤ 6%). This stability is highly important for sample shipment and storage as well as delayed sample taking. As the hair shaft is not subject to biotransformation processes, trimmed as well as untrimmed hair will be valuable specimens for post-exposure analysis targeting long-lasting biomarkers.

## Conclusion

We herein present for the first time a method very well suited for the long-term biomedical verification of SM exposure in hair by µLC–ESI MS/HR MS (PIS) targeting adducts with hard keratins of hair. Three alkylated peptides obtained after proteolysis are presented as valuable biomarkers. As hair is not considered as potentially infectious material, its handling and shipping are possible without the need to adhere to the strict IATA packaging regulations (International Air Transport Association 2021). Accordingly, hair samples are highly valuable specimens as they can easily be taken by any non-specialized person thus not requiring authorized and qualified health personnel with access to appropriate equipment for blood drawing and subsequent centrifugation. This is an important advantage especially in crisis regions with non-optimum medical infrastructure (John et al. 2016).

As SM-induced alkylation of keratins requires the presence of water, collected contaminated hair should be covered with water for at least 20 min prior to sample preparation, thus allowing optimum adduct formation with SM that still might stick to the surface of the hair. The high stability and insensitivity to washing with shampoo makes hair an ideal specimen for biomarker analysis and allowed a longer period of traceability than that already known from HETE-hemoglobin adducts (12 weeks) (Xu et al. 2014). Evidence of exposure might presumably be possible, even though hair might be cut several weeks up to months after exposure. Future studies might transfer the method to hair and wool from animals to expand and improve the toolbox of verification methods of SM exposure.

## Abbreviations

CBB: Coomassie Brilliant Blue
CE: Collision energy
CID: Collision-induced dissociation
CWA: Chemical warfare agent
CWC: Chemical weapons convention
d_3_-Atr: Triple deuterated atropine
ddMS2: Data-dependent tandem-mass spectrometry
ESI: Electrospray ionization
FA: Formic acid
Fwhm: Full width at half maximum
HETE: Hydroxyethylthioethyl-moiety
HEK: Human epidermal keratinocytes
HR MS: High-resolution mass spectrometry
IATA: International Air Transport Association
iPrOH: Iso-propanol
ISVF: Ion spray voltage floating
µLC: Micro-liquid chromatography
LOI: Limit of identification
MS: Mass spectrometry
MS/MS: Tandem-mass spectrometry
MW: Molecular weight
MWCO: Molecular weight cut-off
NMR: Nuclear magnetic resonance
OPCW: Organisation for the Prohibition of Chemical Weapons
pI: Isoelectric point
PIS: Product ion scan
Qual: Qualifier ion
RCF: Relative centrifugal force
RSD: Relative standard deviation
RT: Room temperature
SDS-PAGE: Sodium dodecylsulfate polyacrylamide gel electrophoresis
SM: Sulfur mustard, (bis(2-chloroethyl) sulfide)
t_R_: Retention time
TOF: Time-of-flight
UF: Ultrafiltration
XIC: Extracted ion chromatogram
